# Note on the Utility of Iodoform in the Treatment of Phthisis, and Its Occasional Toxic Effects

**Published:** 1883-12

**Authors:** R. Shingleton Smith

**Affiliations:** Physician to the Bristol Royal Infirmary


### NOTE ON THE UTILITY OF IODOFORM IN THE TREATMENT OF PHTHISIS, AND ITS OCCASIONAL TOXIC EFFECTS. By R. SHINGLETON SMITH, M.D. Lond., B.Sc., M.R.C.P., Physician to the Bristol Royal Infirmary.

THE earliest observations on Iodoform are those of Righini, who maintains that this substance is superior to other preparations of Iodine in possessing anæsthetic as well as antiseptic and antimiasmatic properties. It does not cause the local irritation and other symptoms which result from other preparations of iodine.

The drug was found in the excretions, also in the blood, in saliva, sweat, milk, tears, mucus of nose, menstrual fluid, urine, bile, fæces, and liq. ammii. Emaciation was never observed. It was given in doses amounting to three grammes daily; after very large doses Maitre observed appearances of iodism.*

Attention was directed to this drug by Semmola at the International Congress at Amsterdam,† and his facts are confirmed by others.‡

"Iodoform acts in phthisis and chronic bronchial catarrh as an anæsthetic, antiseptic, and alterative. Incipient phthisis is often cured. In advanced cases expectoration is lessened, cough and fever are moderated, caseation is arrested, and life is prolonged."

"The dose may be increased to six or eight grains daily."

Dreschfeld claims the following benefits for the drug:—

* *Brit. and For. Med. Chi. Review*, July, 1864, p. 247.

† *Lancet*, Aug. 1882, p. 326. ‡ *London Med. Record*, June 15th, 1883.

1. Increase of weight. 2. Increase of appetite. 3. Diminution of cough and expectoration. 4. Diminution or even total cessation of night sweats. 5. Temperature was often a little lowered.

"No symptoms of iodoform intoxication had ever been seen." *

Anæsthetic or hypnotic effects have been observed by Binz, who says:—"Since a yeast cell, when touched by iodine, decomposes no more sugar, and a colourless blood cell sends out no more processes, a brain cell, under the same influence, suspends its peculiar activity; it no longer receives impressions from without, and ceases to produce impulses for its centrifugal filaments. It sleeps, if the condition produced by iodine be reparable; it dies, if its structure has been organically altered."†

Oberlaender reports case of female patient with gummy tumours, who took forty-two grammes during a period of eighty days. She was then attacked by dizziness, weakness, and diplopia, which lasted two days and a half. Afterwards she began to vomit, and fell into a deep sleep; this drowsiness alternated with excitement, foolish talking, feeling of anxiety, and twitching of face, and for twelve days she was unable to stand alone or walk.

Another patient took five grammes in seven days. Somnolence, uncertain gait, and headache were followed by coma which lasted for five days, and the patient was not free from the symptoms for fourteen days.‡

The following case is a remarkable illustration of both the benefits which may be derived from iodoform even in

* *B. M. J.*, April, 1883, p. 817; 1882, Vol. II., p. 169.

† *Arch. Exp. Path. u. Pharmakologie*, Band VIII., p. 310; Band XIII., p. 159.

‡ *Deutsche Zeitschrift für pr. Medicin*, 1878, No. 37.

acute and advanced cases of phthisis, and also of its hypnotic action :—

Wm. C., æt. 35, used to weigh 12st. 4lbs.; six months ago was 10st. 12lbs. ; on June 16th was 9st. 8lbs. ; present weight, August 15th, 1883, 8st. 8lbs. with clothing.

A year ago last June had hooping-cough, and has had cough ever since, but never expectorated any blood; has brought up a little thick phlegm, and perspired freely at night. He believed that his mother and father died of consumption brought on by drink.

Patient was much emaciated, temperature 101° evening; breath short; pulse quick, weak and dicrotous. Dullness at both apices, front and back; the right dull all the way down, left dull to middle of scapula. Crepitation on coughing, but no cavernous sounds. Sputum contained numerous tuberculous bacilli. On August 17 was ordered iodoform, gr. ij., in pil. every four hours.

Aug. 21.—Iodoform increased to three grains; weight, 8st. 1lb. without clothing.

Aug. 28.—It was increased to four grains; weight, 8st. 2lbs. without clothing.

Sept. 4.—It was increased to six grains; weight, 8st. 1lb. without clothing.

Sept. 16.—The urine became of very deep red colour on adding nitric acid, and blue on the further addition of starch. Patient had much improved in general condition. Evening temperature 100°; there was no pain, no perspiration, and he had gained strength. This steady improvement continued up to Oct. 2nd, when he complained of increasing weakness, and on the 3rd he vomited several times; he also complained, on Oct. 5th, that he could not see at all distinctly, everything appeared to be in a fog; he was not able to read, and he saw cats and other creatures running about. He had various delusions and hallucinations, and was so giddy and weak that he was obliged to remain in bed. The eyes were examined, but no abnormality was discovered. The urine contained evidences of iodine, but no albumen. Morning temperature was 98°·0; evening temperature was 99°·2.

As it was presumed that the mental phenomena might be due to the iodoform, which the patient had taken in quantity amounting to thirty grains daily from Sept. 5th, this drug was discontinued on Oct. 6th, and mist. quinæ given three times daily.

On Oct. 7th the sight had improved, and he was more sensible; accordingly on the 10th iodoform was again given in doses of three grains every four hours.

On the 12th he again had hallucinations, he fancied he saw cats, and was incoherent and nonsensical in his conversation; he also again complained of dimness of sight.

On the 16th he still complained of dimness of vision; he could not read the newspaper, although the pupils were not now dilated by atropine or contracted by eserine. The nurse stated that he talked nonsense all the day, and seemed quite strange and changed in character. He had little cough or expectoration, and no pain.

22nd.—He was less weak, and able to sit up all day; slept fairly. There was no delirium or dreaming, but sight still obscured. The iodoform had been discontinued on the 18th, and the urine now contained no trace of iodine. There was copious muco-purulent expectoration, much increased since the discontinuance of the pills.

He was ordered iodoform, gr. iij., twice a day, and ol. morrhuæ, ʒj. ter die.

26th.—He complained of difficulty in walking, his feet felt sore as if from walking on pebbles. There was no œdema, no ankle-clonus, and knee-reflex was normal. The muscles of legs were painful at night. There was still complaint of dimness of sight and want of sleep.

Nov. 2nd.—In consequence of a continuance of drowsiness, soreness of feet, pains in legs, and dimness of vision the iodoform pill was altogether discontinued.

Patient was then ordered Easton's syrup, ʒj. ter die.

6th.—The feet swelled a little at night; they still felt numbed; patient was still very drowsy, and could not see well. Was neither gaining nor losing in weight, and had no active symptoms.

9th.—The sputum was becoming more abundant, and contained crowds of tuberculous bacilli.

This case affords some illustration of the results which are commonly observed in most cases of phthisis as a result of the administration of iodoform. In a large number of cases where I have watched its effects similar results have occurred: the temperature falls, the general strength improves, the expectoration diminishes or ceases entirely, the mischief which was before actively progressive in the lung becomes stationary or retrogressive, the weight of the patient steadily increases, and the bacilli become much less numerous or may even disappear altogether from the expectoration.

When such benefits as these are being derived one is naturally inclined to increase the dose of the drug until some physiological toxic results are manifested, and the case further illustrates the physiological limit beyond which it is not safe to go. This limit probably varies much in individual cases: I have observed indications of iodism in some subjects with one-grain doses of iodoform three times daily, in others there has been a complaint of nausea with a similar dose, but there are very few patients (and one need not exclude children) who cannot take the drug in the ordinary pill form or in the " McK. and R." gelatin capsuled pill. In no case have I given more than six-grain doses five times daily, but I have frequently given five-grain doses and without any deleterious result. It will be observed that toxic symptoms did not manifest themselves until nearly two months after the patient commenced the drug, and not till he had steadily continued with the full dose of six grains five times every twenty-four hours for one month. It will further be seen that the symptoms commenced to abate immediately on the discontinuance of the drug, and were renewed as the drug was again resumed in one-half the previous dose; they then continued as long as the drug was given, and diminished when it was given up. The persistence of the symptoms is remarkable: the iodoform was finally withheld on November 2nd, but the patient's mental condition and his sight are not yet (November 22nd) restored to their normal condition.

The weekly list of weight and average temperature is as follows:—

| | | | | | | | |
|---|---|---|---|---|---|---|---|
| Aug. | 21. | Weight | 113 | pounds. | Average | temp. | 99°·2 |
| " | 28. | " | 114 | " | " | " | 99°·6 |
| Sept. | 4. | " | 113 | " | " | " | 99°·3 |
| " | 11. | " | 112 | " | " | " | 98°·6 |
| " | 19. | " | 113 | " | " | " | 98°·8 |
| " | 25. | " | 113 | " | " | " | 99° |
| Oct. | 3. | " | 110 | " | " | " | 99° |
| " | 10. | " | 109 | " | " | " | 98°·6 |
| " | 17. | " | 112 | " | " | " | 99°·2 |
| " | 24. | " | 115 | " | " | " | 99° |
| " | 31. | " | 115 | " | " | " | 99° |
| Nov. | 7. | " | 115 | " | " | " | 98°·8 |

